# Protein dynamics and structural waters in bromodomains

**DOI:** 10.1371/journal.pone.0186570

**Published:** 2017-10-27

**Authors:** Xiaoxiao Zhang, Kai Chen, Yun-Dong Wu, Olaf Wiest

**Affiliations:** 1 Lab of Computational Chemistry and Drug Design, Laboratory of Chemical Genomics, Peking University Shenzhen Graduate School, Shenzhen, China; 2 Key Laboratory of Functional Molecular Engineering of Guangdong Province, School of Chemistry and Chemical Engineering, South China University of Technology, Guangzhou, China; 3 College of Chemistry and Molecular Engineering, Peking University, Beijing, China; 4 Department of Chemistry and Biochemistry, University of Notre Dame, Notre Dame, Indiana, United States of America; University of Lincoln, UNITED KINGDOM

## Abstract

Bromodomains are epigenetic readers of acetylated lysines that are integral parts of histone tails. The 61 bromodomains in humans are structurally highly conserved but specifically bind to widely varying recognition motifs, suggesting that dynamic rather than static factors are responsible for recognition selectivity. To test this hypothesis, the dynamics of the binding sites and structural water molecules of four bromodomains (ATAD2, BAZ2B, BRD2(1) and CREBBP) representing four different subtypes is studied with 1 μs MD simulations using the RSFF2 force field. The different dynamics of the ZA-loops and BC-loops between the four bromodomains leads to distinct patterns for the opening and closing of the binding pocket. This in turn determines the structural and energetic properties of the structural waters in the binding pocket, suggesting that these waters are not only important for the recognition itself, as has been proposed previously, but also contribute to the selectivity of different bromodomains.

## 1. Introduction

**Fig 1:**
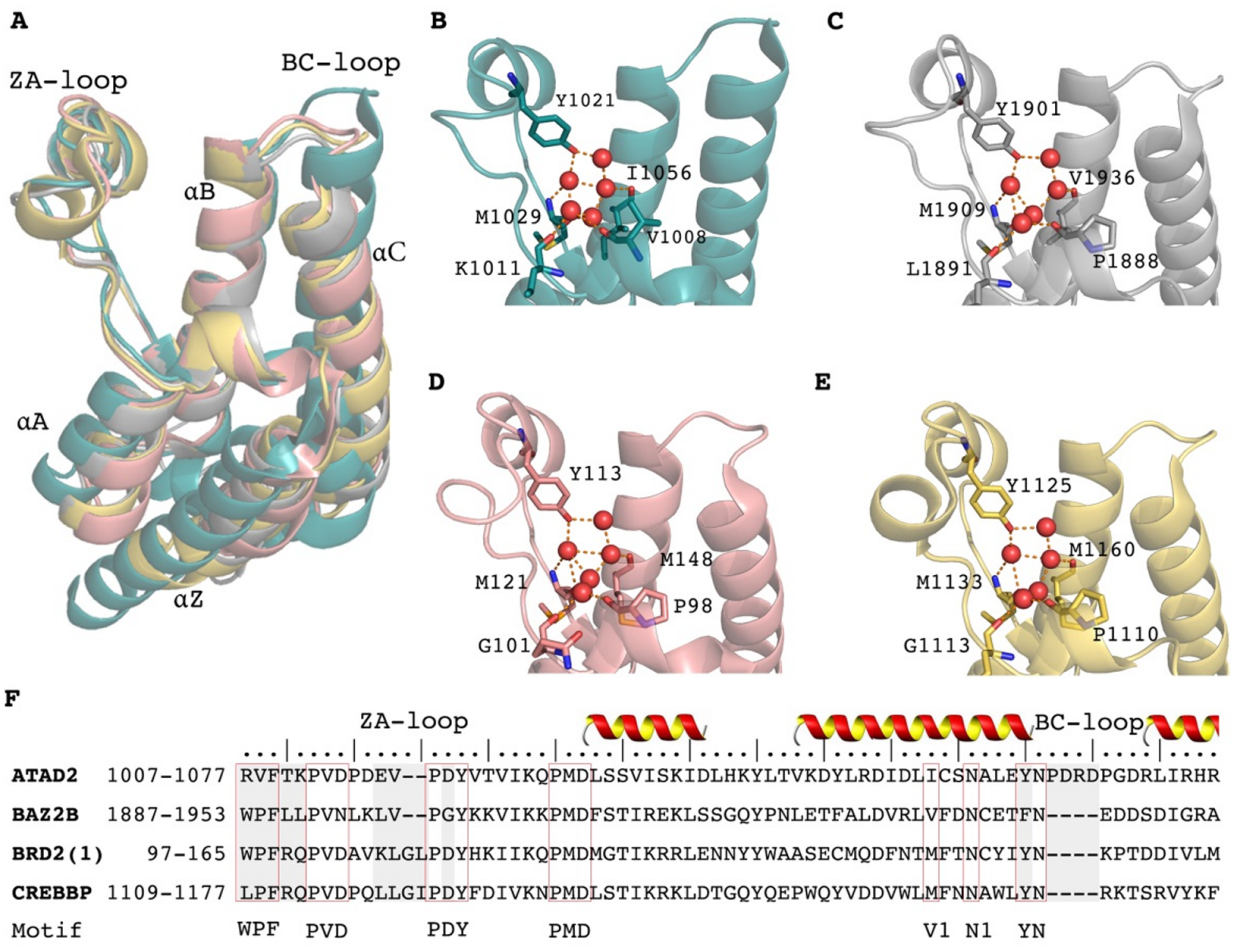
(A) Overlay of apo structures of 4 bromodomains: teal: ATAD2, (3DAI, 1.95Å); silver: BAZ2B (3G0L, 2.03Å); pink: BRD2(1) (1X0J, 1.80Å); gold: CREBBP (3DWY, 1.98Å) (B-F) close-up of binding sites with structural waters shown. (F) Sequence alignment of the four bromodomain pocket from the “WPF” shelf to top of helix C. The grey colored regions show sequence differences that may influence protein dynamics, and the red frames show the conserved motifs (WPF, PVD, PDY, PMD, V1, N1, YN) through the entire bromodomain family, which are used as markers to define the water network.

Lysine acetylation has been found to play a fundamental role during epigenetic regulation of gene expression[1–6]. Bromodomains (BRD) are protein interaction modules that selectively bind ε-N-lysine acetylation (KAc) and other acylation motifs[7]. 61 unique bromodomains, clustered into eight families based on sequence similarity, have been reported to be included in 46 chromatin regulator proteins in humans. Phylogenetic analysis of the human bromodomains identifies eight subclasses, of which subclass II, the bromodomain extra terminal protein (BETs) family are the best studied[8–11]. Since the first three-dimensional structure of bromodomain was solved by NMR spectra in 1999[12], crystal and/or solution structures of over 40 bromodomains with and without bound substrates or inhibitors have been published and released. All bromodomains share a conserved tertiary structure fold consisting of a left-handed four-helix bundle; with the hydrophobic acetyl-lysine binding site at one end of the helix bundle formed by the ZA loop and BC loop (Fig 1A).

The bromodomains shown in Fig 1A–1E not only represent the typical structures of four of the eight phylogenetic subclasses, but are also typical examples for the wide range of biological processes bromodomains are involved in. For example, the C-terminal domain of BRD2 was found to be important for chromatin interaction and regulation of transcription and alternative splicing[13]. I-CBP112, an inhibitor of the subclass III CREBBP, has been found to significantly reduce the leukemia-initiating potential of MLL-AF9(+) acute myeloid leukemia blasts both in vitro and in vivo[14]. Studies show that the subclass IV ATAD2 is a generalist facilitator of chromatin dynamics in embryonic stem cells[15], and that bromodomain mutations can disable ATAD2’s ability of promoting cancer cell proliferation[16]. The function of the subclass V bromodomain BAZ2B is still unclear but it was found that single nucleotide polymorphisms (SNPs) in the BAZ2B gene locus have been associated with sudden cardiac death[17].

In a systematic study of the histone substrates of 43 bromodomains, Filippakopoulos *et al*. concluded that despite the sequence differences such as the ones shown in Fig 1F, the bromodomains share a highly similar three dimensional structure and a highly conserved KAc pocket[18,19]. Nevertheless, different post-transitionally modified sequences of the histones are recognized by specific bromodomains[18] based on differences in the surface charge distribution. This apparent contradiction between high structural similarity vs. highly selective recognition of distinct binders raise the question how the sequence differences in key parts of the binding pocket translate into binding affinity differences[20].

A noteworthy feature of all known bomodomain structures is the presence of well resolved structural waters in the binding pocket[21]. Five water molecules are found to be conserved in many high quality bromodomain crystal structures as an integral part of the acetyl-lysine binding pocket[22]. These water molecules were found to be essential in a druggability analysis of diverse members of the bromodomain family which showed that the absence of these five water molecules decreased the discrimination between bromodomain pockets[22]. Even though some aspects of this study were later questioned[23], there is agreement that the structural waters play an important role in differential binding of acetylated lysine in different bromodomains. One possible approach is thus to view the structural water molecules as a removable and functional part of the protein whose dynamics depends on the overall protein dynamics.

Recognizing that crystal structures are not always sufficient to describe dynamical features[24], the Caflisch group studied 20 bromodomains (CECR2, FALZ, GCN5L2, PCAF, BRD4(1), BRDT(1), BRD3(2), PHIP(2), WDR9(2), CREBBP, EP300, ATAD2, BRD1, BAZ2B, TAF(1), TAF1L(2), TAF1(2), ASH1L, PB1(2), SMARCA4) in a series of molecular dynamics (MD) studies that revealed a surprisingly high heterogeneity of plasticity of the bromodomain pocket[25], but left the nature and origin of heterogeneity uncharacterized. The same group also simulated two bromodomains (BAZ2B and CREBBP) to show that the occupancies of these water molecules were coupled with conformational transitions of the ZA loop, and some of them could be transiently replaced by cosolvent (i.e. dimethylsulfoxide, methanol, or ethanol)[26].

Here, we probe the hypothesis that dynamical rather than static structural differences constitute the key differences between the different bromodomains and that the structural waters are integral parts of the selectivity of the binding process. We will investigate the relationship between protein dynamics and potential water displacement using multiple 1 microsecond MD simulations of four characteristic bromodomains (ATAD2, BAZ2B, BRD2(1), and CREBBP) using the RSFF2 force field to study the structural dynamics of the proteins and the structural waters. This study will contribute insights on the protein-water coupled dynamical features of four characteristic bromodomains and help understand the dynamic differences between them with the long-term goal of understanding the basic biophysical features of the binding event and the origin of selectivity between characteristic bromodomains from different subfamilies.

## 2. Computational methodology

### 2.1. Molecular dynamics simulations

Starting structures for the simulations were built based on the X-ray crystal structures of ATAD2, BAZ2B, BRD2(1), and CREBP (PDB code 3DAI, 3G0L, 1X0J and 3DWY, respectively). Water molecules in the crystal structures were removed except for those within 4 Å of a protein heavy atom. Hydrogen atoms were added and optimized using PLOP[27]. The protonation states of all histidines were checked manually based on the local hydrogen bond network. All aspartic and glutamic acids are negatively charged and all lysines and arginines are positively charged. The BRD2(1) structure 1X0J is set as reference of residue numbering in the four bromodomains we studied according to sequence alignment, the correspondence of residue numbering is shown in Table B in S2 File.

MD simulations were performed with Gromacs[28] (version 4.5.4) using the RSFF2 force field[29,30] for the protein and TIP3P water model[31]. The recently developed residue-specific force fields were found in an independent study to be superior to earlier force fields in the correct reproduction of protein structure and folding[32–34]. The protein was solvated in a rhombic octahedral box with periodic boundary conditions and a distance of 10 Å between the boundary and the nearest protein atoms. Sodium and chloride ions were added to neutralize the simulated system and to approximate an ionic strength of about 100 mM. Long-range electrostatic interactions were computed using the Particle Mesh Ewald method. Non-bonded interactions were truncated at a cutoff of 10 Å. Covalent bonds involving hydrogen atoms were constrained using LINCS algorithm[35]. The time step used was 2 fs, and snapshots were saved every 5 ps. The system was minimized for 10000 steps using a steepest descent algorithm, followed by 1 ns heating process to increase the temperature from 10 K to 300 K and 1 ns of NPT simulation with weak restraints on heavy atoms. The 1000 ns of NPT MD production simulation was performed at 300 K via the v-rescale temperature coupling scheme.

### 2.2. Definition of structural waters

**Fig 2:**
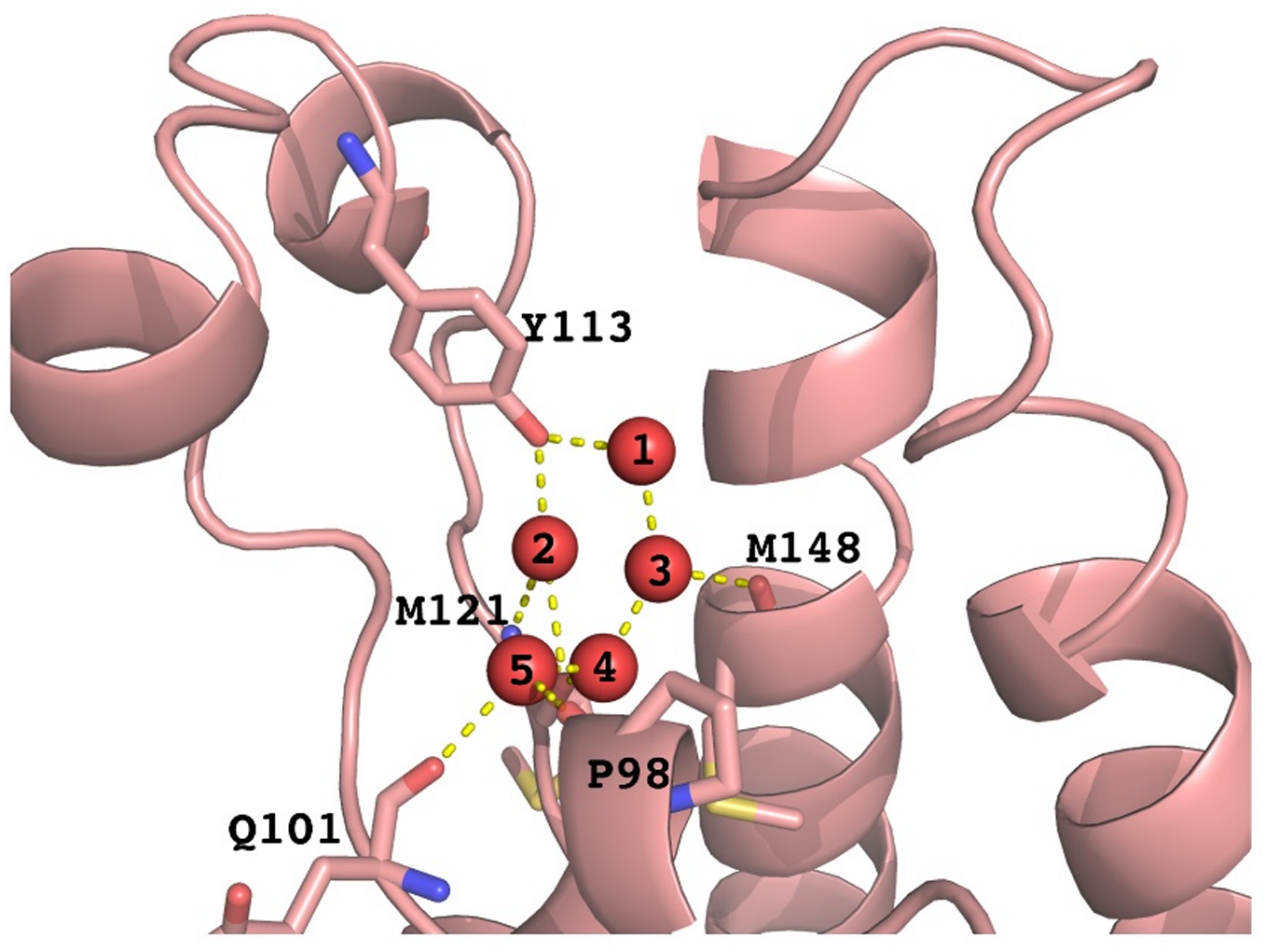
Waters (wat1-wat5, red spheres) at the bottom of the KAc binding pocket of BRD2(1) (PDB code: 1X0J), forming H-bonds with protein residues.

The structural waters and the hydrogen bonding network they engage are defined based on the crystal structures. Fig 2 shows the nomenclature of the waters and the interacting residues of BRD2(1) as example. Wat1 forms hydrogen bonds to Y113, the backbone amide of M121 and the side chain of N151. Wat2 binds the ligand to Y113 in several cases. Wat3 connects wat2 and wat4, and is stabilized by hydrogen bonding with M148. Wat4 forms a hydrogen bond with M121, wat3 and wat5. Wat5 is anchored by the backbone carbonyl oxygen atoms of P98 and Q101. Waters 2 to 5 form a hydrogen bond network at the bottom of bromodomain binding site. In MD simulations, the waters are identified by geometrical measurements (Tables A-B in S2 File).

### 2.3. Properties of structural waters

The thermodynamics of the interaction of the protein with the structural waters was evaluated using two independent analyses: occupancy and replacement free energy. Snapshots representing each trajectory were superimposed to the first snapshot based on cyclical least square fit of C_α_ atoms. Occupancy of waters at position 1–5, as defined by the percentage of each defined water sites being occupied over the course of the trajectory, were calculated.

The structural and energetic properties of the active site waters was calculated using the solvent properties analysis (SPA) method developed by Huang *et al*.[36]. The SPA program computes the replacement free energies of binding site water molecules by post-processing trajectories obtained from MD simulations of the ligand-free protein in explicit water. The water replacement free energy (ΔG_SPA_) is defined as the free energy penalty of removing a specific water molecule from the binding site to the bulk water. The SPA program generates a water oxygen density map over the course of a simulation trajectory by water clustering. Then, SPA calculates the interaction energy water molecules within 4 Å of an identified pocket center based on force field parameters. SPA reproduced the structural properties of waters in 124 crystal structures, along with their energetic properties computed by the double decoupling method. Here, we extract 1000ns trajectories of the four bromodomains with one frame per 200ps to calculate water clusters and replacement free energies.

## 3. Results and discussion

### 3.1. Dynamics of bromodomains

To understand differences in the dynamics of the four bromodomains, the binding pocket fluctuations as a function of the protein sequence were studied. The binding pocket can be divided into several segments, including conserved and specific binding hotspots. The superimposition of static crystal structures (Fig 1A–1E) shows that the four bromodomains share the similar pocket shapes and water-bound residues, while the sequence alignment reveals four major sequence differences that needed to be considered (Fig 1F): (a) the WPF sequence that forms a shelf on the binding site in BAZ2B and BRD2(1) is replaced by a RVF shelf in ATAD2 and the LPF sequence in CREBBP. (b) the ZA-loops in ATAD2/BAZ2B are two residues shorter than in the other BRDs. (c). the PDY-YN residue that anchor waters changes into PGY-FN in BAZ2B, causing removal of the H-bonding interaction and weaken of the stacking interaction. (d). the helix C is longer in ATAD2 than that in the other BRDs. With these differences in mind, analysis of the 1 μs trajectories can provide insights into the dynamic consequences of these sequence differences.

**Fig 3:**
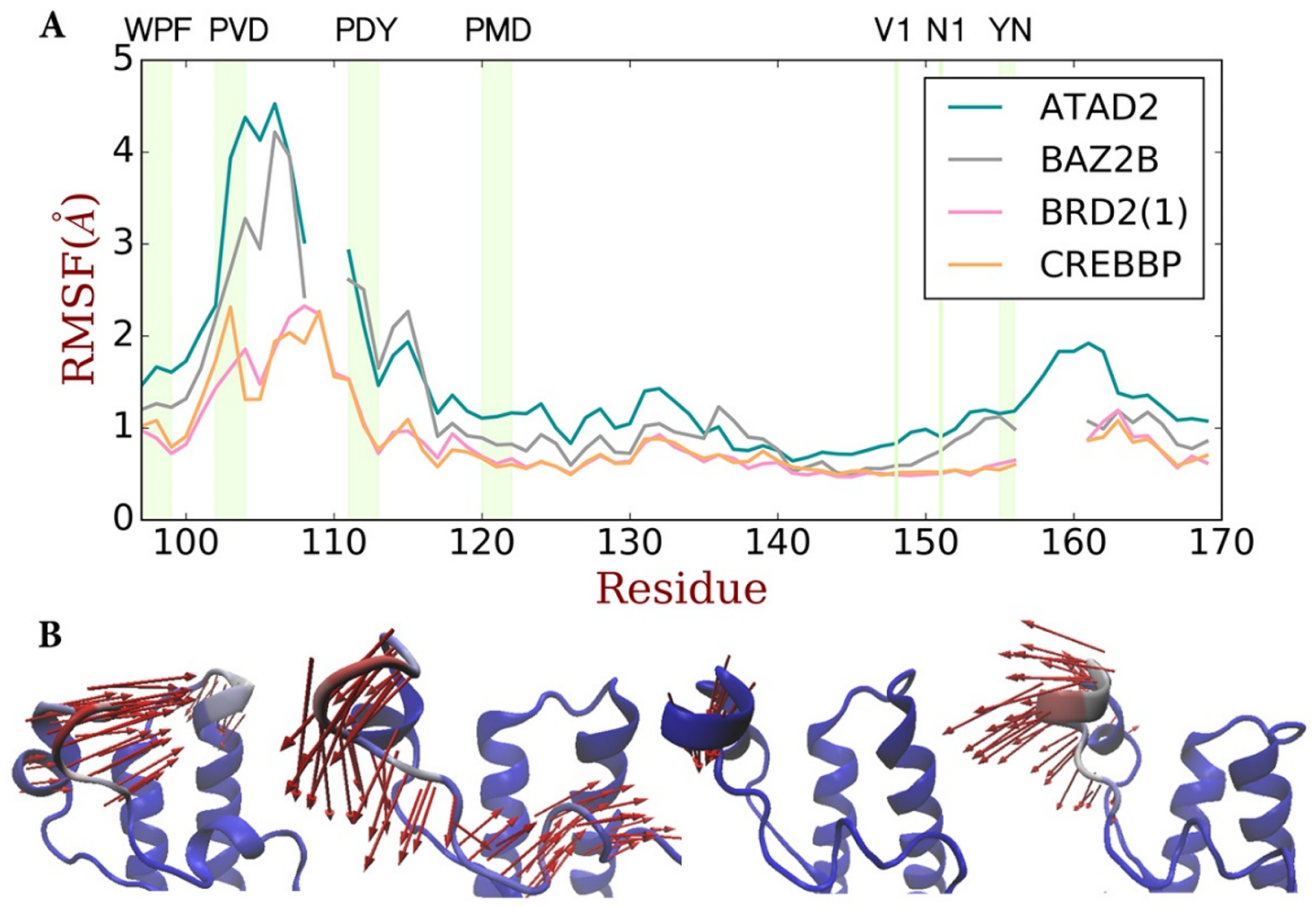
(A) RMS fluctuation plot of the bromodomain pocket. The fluctuations of absent sequences are not shown. Fluctuations of the conserved markers (WPF, PVD, PMD, V1, N1, YN) are labeled and colored with light green background. Residue numbering is based on the BRD2(1) (PDB code: 1X0J). (B) The first principal component of dynamics from bromodomain simulations imaged by VMD[37]. From left to right, the bromodomains (ATAD2, BAZ2B, BRD2(1) and CREBBP) are shown in cartoon, colored from red to blue indicating increasing flexibility. The green arrows show the direction of specific residue fluctuation and point out major differences between the four bromodomains.

The Cα Root mean square fluctuation of each residue in the MD simulation of the four bromodomains shown in Fig 3A indicates two peaks representing the PVD-PDY segment (specifically L108) and the BC-loop, responsible for ligand binding that are significantly different between ATAD2 and BAZ2B on one hand and the much less flexible remaining three BRDs on the other. In ATAD2, the most flexible region is around PVD motif on the ZA-loop, which is in good agreement with the previously postulated observed induced fit during ligand binding[38,39]. The second flexible region is the BC-loop and the top of the Helix C consisting two Asp residues, which is involved in selective ligand binding[40]. The RVF shelf of ATAD2 is as stable as the WPF shelves in BRD2(1) while the flexible regions of BAZ2B are P1888-Y1901 on ZA-loop and “DDSD” on BC-loop. These results are in good agreement with the NMR experiments by Ciulli *et al*. who showed BAZ2B has a relatively rigid core while F1889, Y1901 and D1946 show fast internal motions[41]. The per-residue C_α_ RMSF is also in good agreement with the B-factors from the crystal structures and the mobility of the individual residues in the four bromodomains as deduced from the position of the residues in the different available crystal structures (Fig E in S1 File), suggesting that the simulations are a reasonable representation of the experimentally observed dynamics.

The results shown in Fig 3 provide a more comprehensive and detailed view of these dynamics of the four bromodomains compared to the crystal structures. The fluctuation modes of BRD2(1) and CREBBP are quite similar, but distinct from ATAD2 and BAZ2B, with one flexible region P86-L110 and another flexible region on top of Helix C. In all four bromodomains, the “WPF shelf” and PMD motifs are kept stable in simulations, which is in agreement with experimentally observed hinge consisting of residues P1012 to P1028 specific to ATAD2[39].

Although the magnitude of the RMS flexibility is different, the residues involved in the flexible regions in the four bromodomains are basically similar. Closer analysis of the movement by principal component analysis (PCA), shown in Fig 3B, reveals significant differences in the directionality of the fluctuation of the protein residues and emphasize the distinct pocket shape changes for each of the four BRDs. In ATAD2, the ZA-loop moves vertically and away from BC-loop. As will be shown later, this opening movement breaks the network of structural waters. In BAZ2B and CREBBP, the PVD motif is moving towards the pocket, leading to a “breathing” opening and closing of the binding site. In BRD2(1), the PVD motif is quite stable and the pocket shape volume is fairly constant. In summary, the three dimensional vectors from the PCA describing the fluctuations of the four bromodomains show significant difference in ZA-loop fluctuations and the associated changes in pocket shape and volume, supporting the notion that the differences between the bromodomains, which could rationalize their differences in selectivity and function, are more rooted in the dynamics of protein motion rather than the static structures.

### 3.2. Quantification of pocket fluctuations

**Fig 4:**
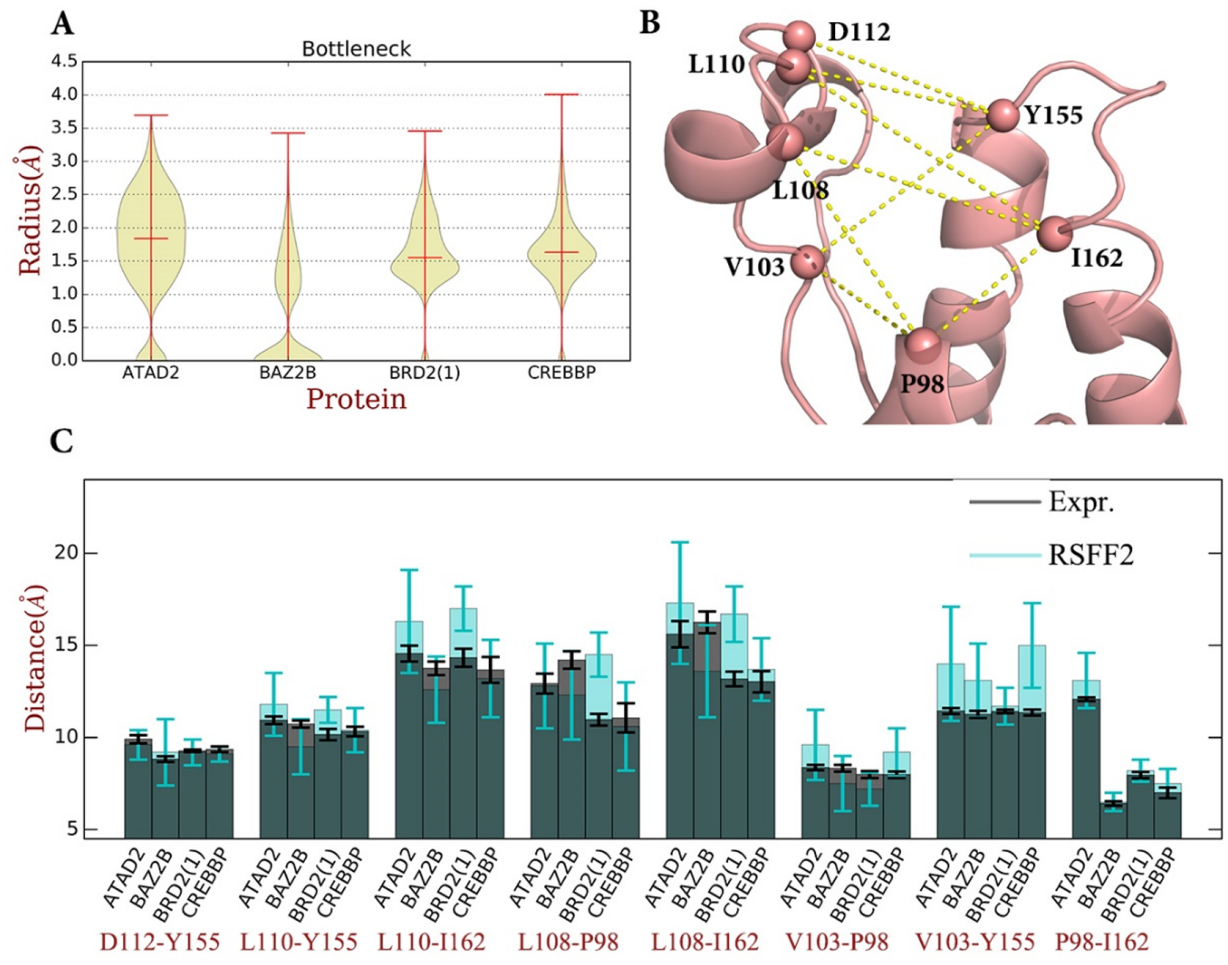
(A). Tunnel radius as calculated by Caver 3.0[42]. Distributions of the bottleneck radius over the 1 μs trajectory for each BRDs are shown in violin plot. (B) The C_α_ atoms of key residues for BRD2(1) structure (PDB code: 1X0J) with distances used in gold. (C) Distance distribution for eight distances between key residues (D112, Y155, L108, L110, V103, I162, P98) around the BRD pocket. Average and standard deviations from different conformations in the unit cell for holo- crystal structures and from 1 μs trajectories for MD simulations are shown in grey and cyan bars, respectively.

To quantify the bottleneck residue fluctuations, ligand accessibility in the structures from the MD simulations was calculated as the radius of the tunnel leading to the binding pocket (Fig 4A, Figs. F and J in S1 File) using Caver 3.0 software[42], which is widely used for the identification and characterization of transport pathways in macromolecular structures. Caver 3.0 identifies tunnels in protein conformational ensembles and output time evolution of individual tunnels. This tunnel was found to be delineated mainly by the PVD, PDY, PMD and YN motifs (V 103, Y113, M121, Y155, N156 in BRD2(1)), with the bottleneck being determined by the dynamics of the PVD and PDY motifs (Fig J In S1 File). The bottleneck radius dynamics is thus strongly correlated with the dynamics of the PVD dynamics. As shown in Fig 4A, the bottleneck radius can be as small as 1Å in extreme cases, which is too small to allow the entry of a water molecule. Consistent with the results discussed above, the bottleneck of ATAD2 has a wider distribution indicating higher flexibility while the other three pockets have an average bottleneck radius around 1.6 Å and a narrower distribution.

To quantitatively describe the pocket shape itself, 7 key residues around the KAc binding pocket shown in Fig 4B were used: Y155 and I162 on helixes, D112 and P98 of medium fluctuation on ZA-loop, V103, L108 and L110 of the highest fluctuations on ZA-loop. Eight distances between the Cα atoms of the markers that reflect the backbone dynamics for the depth and width of the binding pocket, were selected: D112-Y155, L110-Y155, L110-I162, L108-P98, L108-I162, V103-P98, V103-Y155 and P98-I162, respectively. Of these, L108 and L110 are of particular interest because analysis of the crystal structures used here as well as many others in the PDB indicates that they are often involved in hydrophobic interactions with binders. At the same time, distances to L108 and L110 show the largest fluctuations in all four bromodomains as shown in Fig 4C, even though the three dimensional vectors are quite distinct as shown previously.

To compare these computational results to experimental data, we performed a comprehensive analysis of the available crystal structures (see Fig B in S1 File and Table F in S2 File). Search of the protein database for holo structures of bromodomains with resolutions of better than 2.1 Å showed 65 entries, with several entries having non-identical monomers in the unit cell leading to 105 unique structures. Alignment and statistical analysis of distances in the unique structures then provides the distribution of the key parameters that define the pocket shape and the structural water occurrence discussed below. Fig 4C shows the comparison of the pocket shape of the holo crystal structures as defined by the eight distances with that of the simulation trajectories for the four bromodomains. In the available crystal structures of the BRDs bound to inhibitors, (dark grey in Fig 4C), D112-Y155, L110-Y155, L110-I162, V103-P98 and V103-Y155 are essentially identical between the different BRDs while the three other parameters show significant differences between them. Specifically, the shorter ZA-loops in ATAD2 and BAZ2B cause increases of the L108-P98 and L108-I162 distances, indicating that the pockets are more open. The longer helix C in ATAD2 causes a 5Å increase in the P98-I162 distance, indicating a deep pocket, while in BAZ2B this distance decreases because of a shorter helix C. In summary, BRD2(1) and CREBBP share similar pocket shapes, while ATAD2 has a deeper and more open, hydrophilic pocket, and BAZ2B has a more flat and shallow pocket.

The inherent flexibility of unbound BRDs is difficult to assess experimentally because there are relatively few apo-structures available. 1 μs MD simulations of the apo-structures (light blue in Fig 4C) thus provide insights not easily available otherwise. The differences between the crystal structures and MD simulations for D112-Y155, L110-Y155, and P98-I162 are relatively small. Nevertheless, it is clear that even these distances are more dynamic in ATAD2 and BAZ2B, again in good agreement with the interpretation of Ladbury *et*. *al* that the apo form ATAD2 is more open than holo ones[39]. In comparison, BRD2(1) is quite rigid with low variation of the distances over the simulation time.

### 3.3. Water dynamics

**Fig 5:**
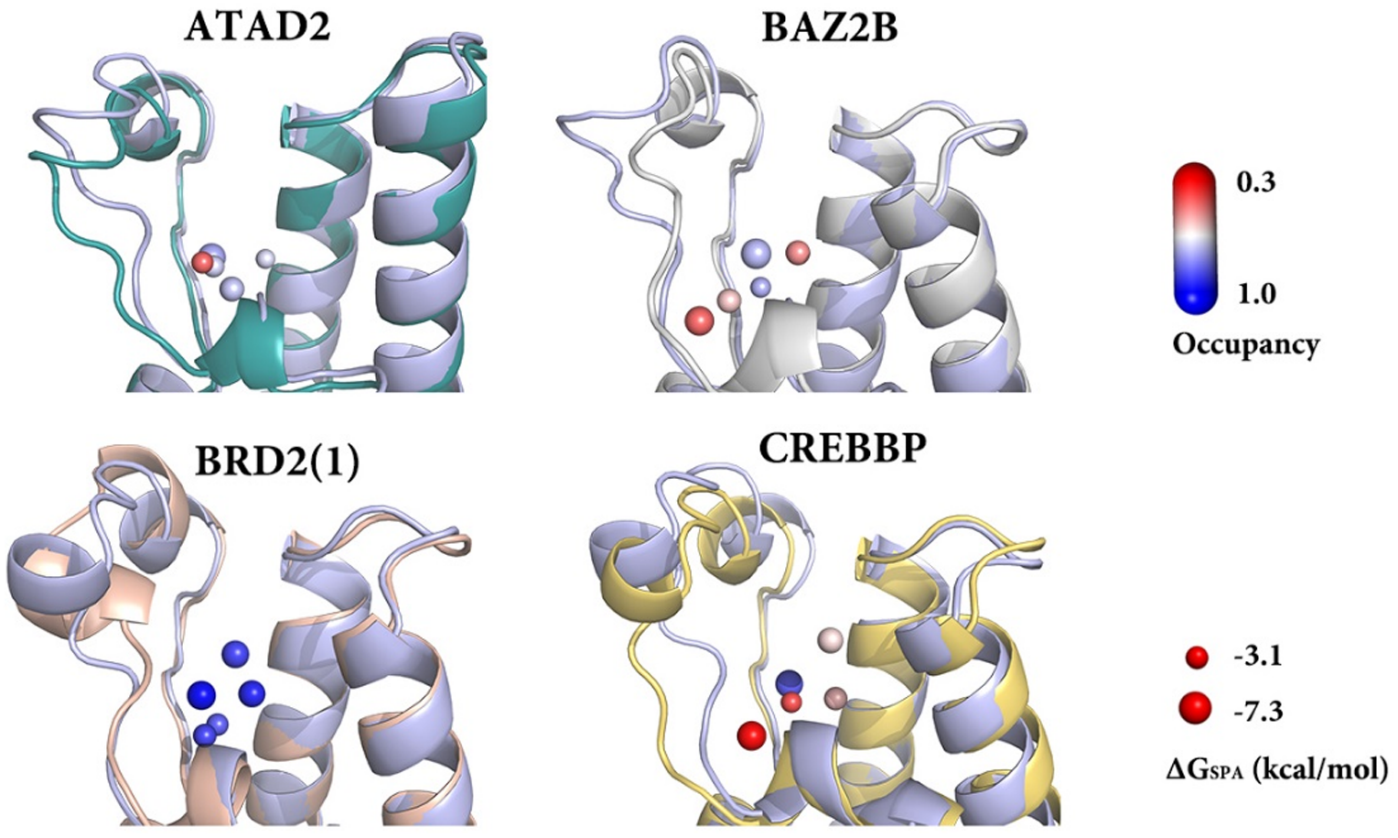
Initial structures of simulations used as reference in light blue and calculated average structure of four bromodomains (ATAD2: teal, BAZ2B: silver, BRD2(1): pink, CREBBP: gold) aligned to reference structures. The five water clusters are shown in spheres, scaled and colored based on their occupancy and ΔG_SPA_.

As outlined previously, the five structural water molecules are best considered as an integral part of the binding site and an understanding of their dynamics is not only desirable from a basic biophysical point of view, but a selective displacement of water molecules in some BRDs but not in others could for example be exploited for the design of selective binders. To study the structural and energetic properties of the conserved waters, the occupancy and replacement free energy (ΔG_SPA_) of each structural waters was calculated (Table 1 and Tables B and D in S2 File). The structural results from these 1 μs simulations from clustering of the water positions also summarized in Fig 5.

**Table 1:** The calculated occupancy (Occ) and replacement free energy (ΔG_SPA_) of each structural water in four bromodomains.

Protein	Parameters	Wat1	Wat2	Wat3	Wat4	Wat5
**ATAD2**	Occ (%)	75.4	67.3	70.0	68.0	41.7
	ΔG_SPA_(kcal/mol)	-6.7	-3.1	-4.8	-3.6	-3.4
**BAZ2B**	Occ (%)	72.3	47.2	73.3	55.6	41.9
	ΔG_SPA_(kcal/mol)	-7.3	-4.4	-5.6	-4.8	-4.8
**BRD2(1)**	Occ (%)	98.7	89.4	93.5	84.5	88.5
	ΔG_SPA_(kcal/mol)	-6.5	-5.6	-5.3	-3.6	-3.8
**CREBBP**	Occ (%)	87.2	57.6	54.0	38.7	29.0
	ΔG_SPA_(kcal/mol)	-6.6	-4.8	-4.5	-3.3	-6.3

From the results in Fig 5 and Table 1, it is clear that wat1 has the most negative replacement free energy due to the highly electrostatically favorable hydration site formed by protein residues. This is in agreement with the importance of the interaction with Y113, which has been discussed previously as important for ligand binding in bromodomains[22,25]. In addition, the calculated ΔG_SPA_ is very similar between the different BRDs and over the course of the simulation, in agreement with the deeply buried nature of wat1 and its highly conserved water network. In comparison, wat2-5 are less strongly bound due to fewer contacts with the protein and more access to other waters (Fig 2 and Table B in S2 File), where electrostatic interactions with waters dominate the free energy, and the differences between the different BRDs is more pronounced, as will be discussed in more detail below. It is also noteworthy that the SPA analysis also identified a water cluster bound to N156 with ΔG_SPA_ of ~2–4 kcal/mol (Table D in S2 File), indicating that displacing the N156 bound water is easier than displacing one of the five conserved waters. This is consistent with experimental observations that N156 act as inhibitor anchor by forming an H-bond replacing a bound water molecule[43,44], validating the results by suggesting that SPA is able to identify replaceable waters in bromodomains.

Combining the calculated occupancy over the course of the 1 μs simulation with the energetic results for ΔG_SPA_ reveals some interesting differences between the individual bromodomains and waters. Consistent with the previously described conformational rigidity of BRD2(1), the water network is well preserved with occupancies mostly larger than 85%. The replacement free energies show that wat4 and wat5 are less favored compared to wat1-3, but wat5 is deeply buried and would require significant rearrangement of the protein to be replaced, as indicated by its high occupancy. As a result, the design of binders that replace wat1 in BRD2(1) is predicted to be difficult while even replacing the most weakly bound water molecule, wat4, is unlikely to yield potent binders.

The results in Fig 5 and Table 1 indicate that the water networks in ATAD2, BAZ2B, and CREBBP, for which much fewer experimental data are available, are distorted to different extents. Wat3 is exposed to the water environment while forms H-bond with M148_O on helix C. Thus, wat3 shows moderate occupancy, ΔG_SPA_ in all the four bromodomains. Most notably, wat5 occupancy in ATAD2, BAZ2B and CREBBP is significantly lower than that in BRD2(1), followed by wat2 and wat4 as the easiest to be displaced from both structural and energetic points of view. Although these differences in water occupancy between the different bromodomains raise the intriguing possibility of achieving selectivity by selective displacement of waters, there are unfortunately not enough crystal structures of holo complexes of ATAD2, BAZ2B and CREBBP available to explore this hypothesis based on experimental data. However, it is noteworthy that in one of the few available holo-structures of ATAD2 (pdb: 4TZ2, chain A) complexed with a very weak binder, wat2-wat4 but not wat1 are replaced by a benzene ring. As shown in Table C in S2 File, displacement of wat2-4 are also observed in crystal structures of other bromodomains, especially in BRD4(1), PB1(5) and TAF1(2). At the same time, wat5, though weakly bound, is not replaced. It can be hypothesized that this is because this view of water binding neglects the fact that replacement of any of the waters by a binder requires a rearrangement of the protein itself. This would have a significant effect on the deeply buried water wat5. It is therefore clear that for a more complete picture, the coupling of protein and water dynamics needs to be considered.

### 3.4. Relationship between protein and water dynamics

**Fig 6:**
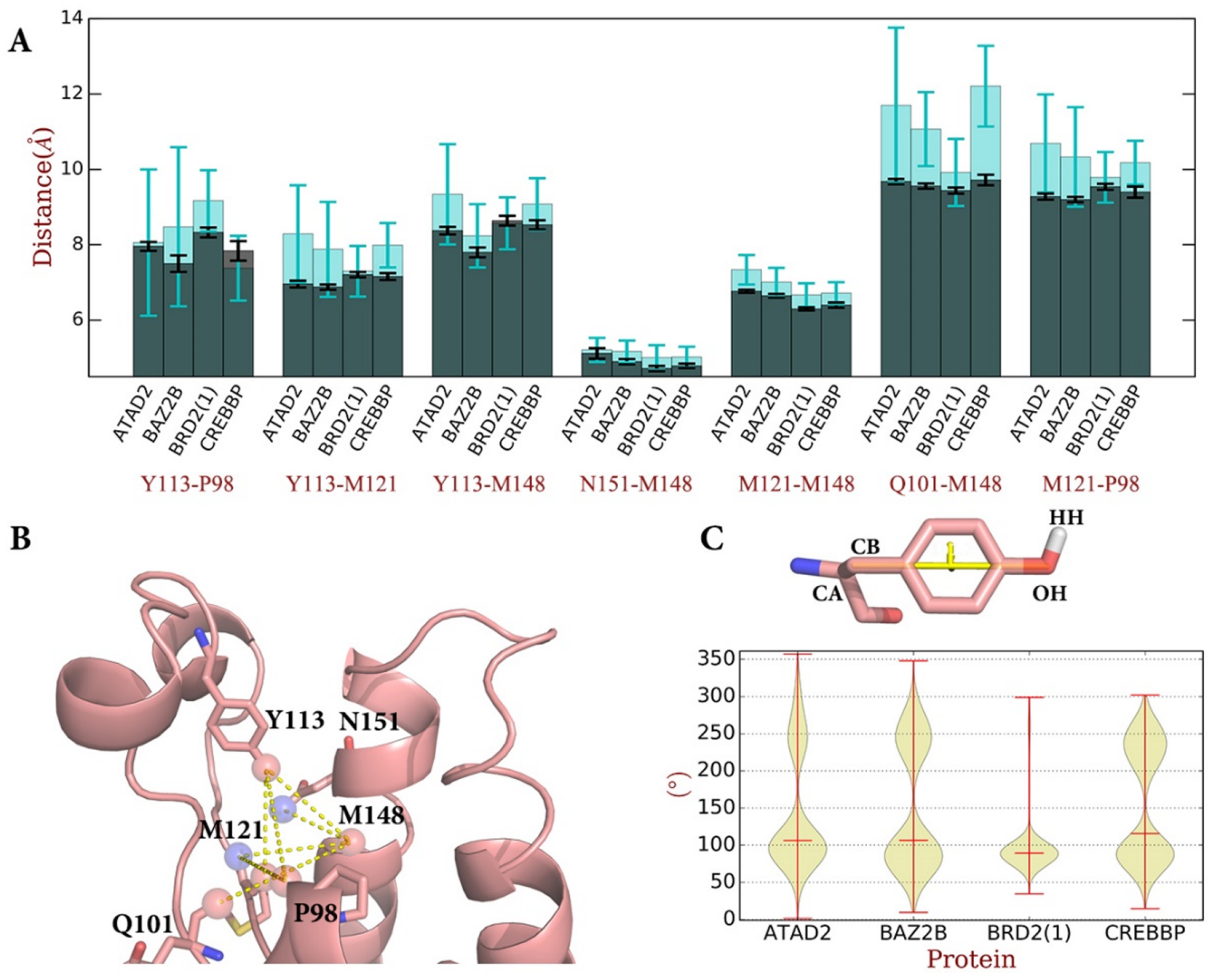
(A) 7 markers (Y113, P98, M121, M148, N151, M121, Q101 in BRD2(1)) directly involved in water network are chosen and 7 distance between the markers are measured. The average and standard deviation are calculated in both 105 holo crystal structures and in MD simulations, shown in grey and cyan bars and error bars, respectively. (B) The markers are shown in red (oxygen) and blue (nitrogen) spheres on BRD2(1) structure shown in pink cartoon using PyMOL[45] (PDB code: 1X0J). (C) The Y113-O dihedral is defined as CA-CB-OH-HH dihedral. The distributions of Y113-O in four bromodomains are shown in violin plot, indicating distinct features. In BRD2(1) the residue is kept in favorable conformation instead of fluctuations observed in ATAD2, BAZ2B and CREBBP.

Based on the hydrogen bond between the conserved Y113 to wat1 and wat2 as well as the hydrogen bonding network among the waters, we hypothesized that the dynamics of ZA loop, containing Y113 on one side of the binding site, and the WPF shelf on the other will have the biggest effect on the water dynamics. In analogy to the approach used earlier, these interactions were mapped by calculating seven distances between atoms which can form direct H-bonds with the structural waters over the course of the 1 μs simulation of the apo structures and comparing the results to the available crystal structures from the statistical analysis discussed earlier (Figs A and G in S1 File). The distances chosen were: Y113-OH_P98-O, Y113-OH_M121-O, Y113-OH_M148-O, N151-ND2_M148-O, M121-N_M148-O, Q101-O_M148-O, M121-N_P98-O and are shown in Fig 6B. These were chosen because in the holo- structures, there are only slight differences between the distance averages in the four bromodomains, as shown in Fig 6A. Thus, the influence of sequence variation has been reduced around the conserved water-residue module, allowing a less biased analysis of the effect of the dynamics. As shown in Fig 2 and Fig C in S1 File, the main interactions between the protein and the waters are the hydrogen bonds of wat1 with Y113 and M121, wat2 with Y113 (but not N151), wat3 with M148, wat4 with M121 and wat5 with P98 and Q101. The dynamics of the waters will therefore be affected by the movements of these residues.

It is clear from the results shown in Fig 6A that the MD simulations provide a more complete picture of the dynamics of the binding site. The M148-O and N151-ND2 atoms are located on the helix and the corresponding distances N151-ND2_M148-O and M121-N_M148-O are tightly distributed for all four bromodomains in both the MD simulation and the crystal structures. This indicates the rigidity of this side of the binding site and, in agreement with the results shown in Fig 5, wat1 and wat3 for which M148 and N151 are structural anchors.

In contrast, the collective motion of the ZA-loop affects the entire water network. As shown in Fig 6A, the distances that involve Y113 show a considerably larger variation in the MD simulations of up to ± 2 Å even if the average value is close to the experimentally observed value, e.g. in the case of BRD2(1). Similarly large fluctuations are observed for the other markers that span the binding cleft, e.g. Q101-M148 or M121-P98, which for hydrogen bonds with wat5. It is interesting to note that the deviations from the crystallographic values vary between the four bromodomains, again emphasizing that although the differences between markers in the crystal structures are small, the dynamic movements distinguish the four bromodomains. For example, in ATAD2, BAZ2B and CREBBP, Q101-O_M148-O distance increases, indicating a movement away from this helix. In BRD2(1), the distances fluctuate much less, with no bias towards either an open or close conformation, stabilizing the water network.

Having established that Y113 serves as a key anchor for the hydrogen bonding network that leads to significantly different dynamics in BRD2(1) on one side and ATAD2, BAZ2B and CREBP on the other, we investigated the structural origin of these differences by analyzing the position of Y113 in more detail. For this purpose, we defined one specific dihedral angle to characterize the conformational changes in Y113: Y113-O (defined as CA-CB-OH-HH, Fig 6C). As discussed earlier, Y113 forms two hydrogen bonds with wat1 and wat2, and the Y113-O dihedral from the crystal structure analysis is ~80°. However, it should be kept in mind that the results from the crystal structures are time averages. Indeed, analysis of the MD simulations indicate that the Y113-O dihedral mainly samples two regions: 50°~100° and 210°~260°, depending which water molecule the HH atom of Y113 is pointing to.

Analysis of the relative population of these conformations, which determine the water network stabilization, indicate some interesting differences between the bromodomains that correspond to the conformational rigidity discussed above. As shown in Fig 6C, the Y113-O dihedrals are distributed around 80° in BRD2(1), a position favorable for binding of both wat1 and wat2 (Table 1). In ATAD2, BAZ2B and CREBBP, the Y113-O dihedrals oscillate between the two conformations frequently, indicating an unstable water network. This rationalizes the differences in occupancy for wat1 and wat2 for these bromodomains shown in Fig 5 and Table 1. Cochran et. al discovered that TAF1(2) and TAF1L(2) are capable of accommodating unusual histone acyl modifications (butyryl- and crotonyllysine) and designed ligands by displacement of pocket bottom water molecules, while BRD4(1) binds less potent to these markers because it keeps the water network[7,46]. This selectivity in ligand binding between different bromodomains further supports the computational findings that selective displacement of waters based on differences in protein dynamics could contribute to the selective recognition of natural and non-natural bromodomain binders.

## 4. Conclusions

Using a combination of experimental data from a statistical analysis of 105 unique structures from the pdb and 1μs MD simulations of four characteristic bromodomains, the origin of the apparent dichotomy between the high structural similarity and the highly selectivity of binder recognition is attributed to differences in the dynamic behavior of the protein and the structurally conserved waters as well as the coupling between them. Dynamic fluctuations of the PVD and PDY motif that control the bottleneck of ligand binding tunnel lead to different populations of open and closed conformations for the different bromodomains. In BRD2(1), the ZA-loops are conformational rigid and predominately populate the closed conformation while in ATAD2, the ZA-loop is flexible and with a higher population of the open conformations. Both conformations interconvert frequently in BAZ2B and CREBBP, with the BAZ2B pocket most often narrow and shallow. Thus, these structurally similar bromodomains covering four of the eight subclasses show distinct dynamic features.

The conserved waters also show distinct dynamical features between the bromodomains. BRD2(1) keeps a rigid and well-arranged water network; while in other three bromodomains the waters are distorted due to conformational flexibility, which indicates easier displacement. For example, in ATAD2 wat2 is energetically easiest to be displaced, and the second choice could be wat4 and wat5, whereas wat1 is deeply buried and inaccessible. While the available experimental data on water displacement is limited, it is in good agreement with the computational results.

Beyond a better understanding of the structural and dynamic reasons for the experimentally observed differences between bromodomains, the results also provide a roadmap for the design of future selective inhibitors exploiting the inherent conformational bias of different bromodomains and its consequences for the network of conserved waters.

## Supporting information

S1 File Figures for protein structures, analysis and trajectories. A. Multiple sequence alignment of the four bromodomain; B. Flow chart of survey on bromodomain holo crystal structures; C. RMSD in simulation trajectories; D. Total Energy in simulation trajectories; E. C_α_ RMSF vs. B-factors and crystal structure ensemble displacement; F. Bottleneck radius in simulation trajectories; G. Conserved water network; H. Structures of bromodomains where binding site waters are displaced; I. pdY_O dihedral angle in simulation trajectories; J. Tunnel of BRD2(1) KAc pocket.
(DOCX)

S2 File Tables with pdb structure codes and results for water analysis. A. Crystal and NMR structures used in MD simulation; B. Definition and residue numbers of structural waters; C. Observed cases of replaced (pink) and kept (blue) water molecules in crystal structures of the bromodomains; D. SPA results for water binding; E The percentage of the transitions of water 1–5; F. PDB codes of crystal structures used in the survey of water network.
(DOCX)

S3 File Zipfile with parameters and analysis scripts. These scripts can also be found at https://github.com/XiaoxiaoZhangOuc/PLOS1_BRD.
(ZIP)

S4 File Crystal structure survey. (XLSX)
