# Supplementary material for: Protein dynamics and structural waters in bromodomains
Source: PLoS One. 2017 Oct 27;12(10):e0186570. doi: 10.1371/journal.pone.0186570 (PMC5659604; doi:10.1371/journal.pone.0186570)
Supplement: S1 File — A. Multiple sequence alignment of the four bromodomain; B. Flow chart of survey on bromodomain holo crystal structures; C. RMSD in simulation trajectories; D. Total Energy in simulation trajectories; E. Cα RMSF vs. B-factors and crystal structure ensemble displacement; F. Bottleneck radius in simulation trajectories; G. Conserved water network; H. Structures of bromodomains where binding site waters are displaced; I. pdY_O dihedral angle in simulation trajectories; J. Tunnel of BRD2(1) KAc pocket. (DOCX) [file pone.0186570.s001.docx]

Supporting Information for:

Protein Dynamics and Structural Waters in Bromodomains

Xiaoxiao Zhang,^1^ Kai Chen,^1,2^ Yun-Dong Wu,^1,4*^ Olaf Wiest^1,3*^

1 Lab of Computational Chemistry and Drug Design, Laboratory of Chemical Genomics, Peking University Shenzhen Graduate School, Shenzhen 518055, China

2 Key Laboratory of Functional Molecular Engineering of Guangdong Province , School of Chemistry and Chemical Engineering, , South China University of Technology, Guangzhou 510640, China

3 Department of Chemistry and Biochemistry, University of Notre Dame, Notre Dame, Indiana 46556-5670, USA

4 College of Chemistry and Molecular Engineering, Peking University, Beijing 100871, China

owiest@nd.edu

Figure A. Multiple sequence alignment of the four bromodomain S2

Figure B. Flow chart of survey on bromodomain holo crystal structures S2

Figure C. RMSD in simulation trajectories S3

Figure D. Total Energy in simulation trajectories S3

Figure E. C_α_ RMSF vs. B-factors and crystal structure ensemble displacement S4

Figure F. Bottleneck radius in simulation trajectories S4

Figure G. Conserved water network S5

Figure H. Structures of bromodomains where binding site waters are displaced S6

Figure I. pdY_O dihedral angle in simulation trajectories S6

Figure J. The tunnel of BRD2(1) KAc pocket S7


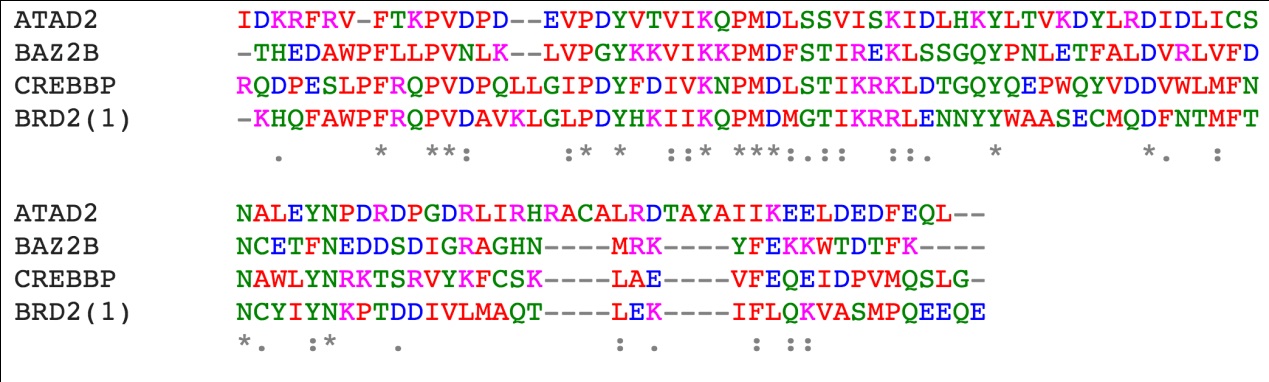


Figure A. CLUSTAL O (1.2.1) multiple sequence alignment of the four bromodomains (ATAD2, BAZ2B, BRD2(1), CREBBP).


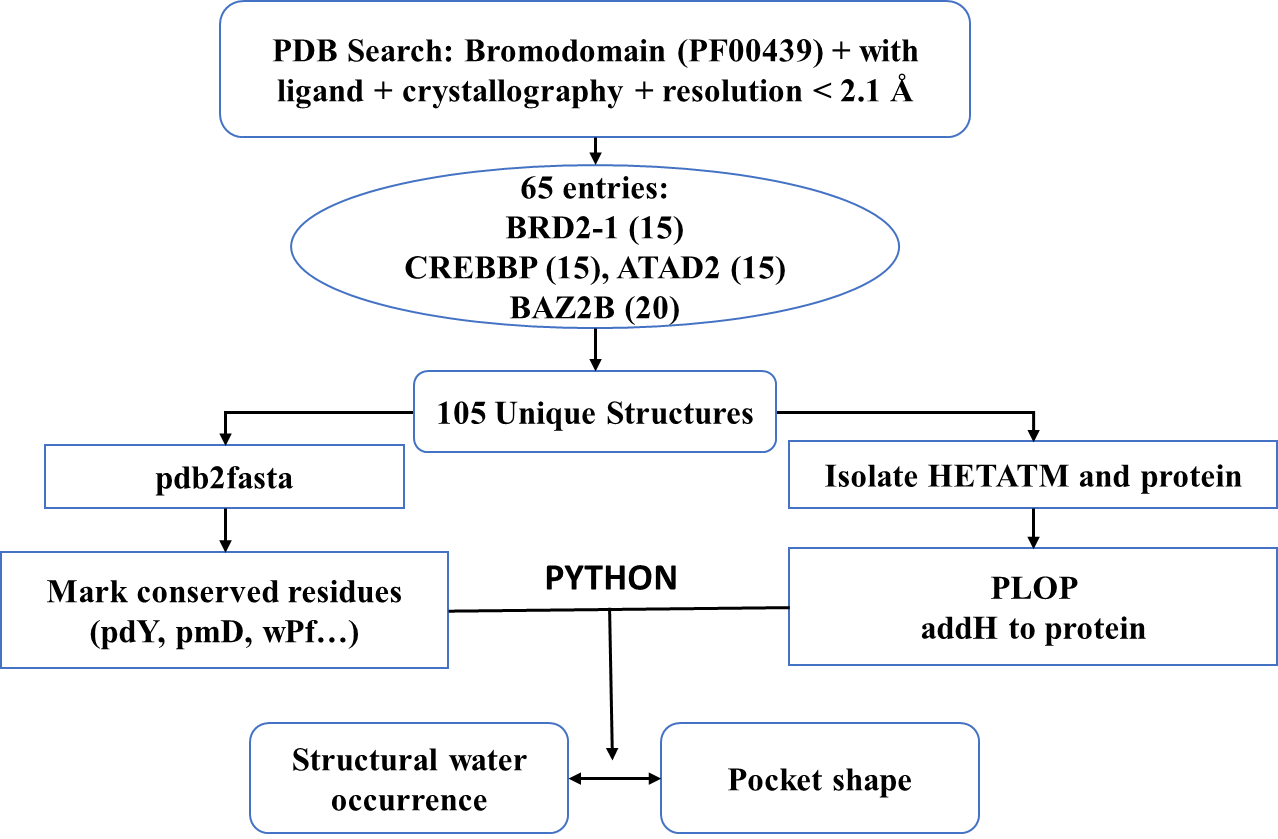


Figure B. Flow chart of survey on bromodomain holo crystal structures, especially on the 4 popular bromodomains, the analysis mainly bases on PLOP program and python scripts to investigate the structural water occurrence and pocket shape.

All scripts used for the crystal structure and trajectory analysis can be found in S3 File and were also deposited at https://github.com/XiaoxiaoZhangOuc/PLOS1_BRD.


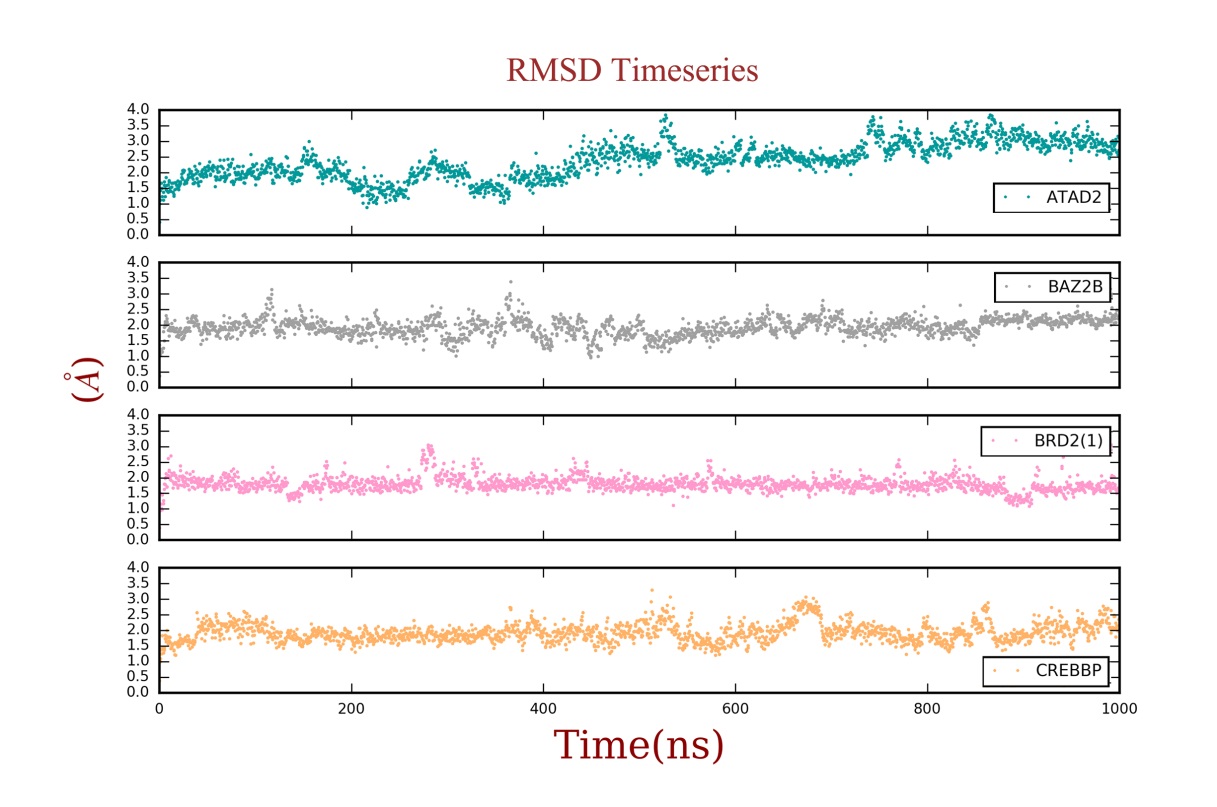


Figure C. RMSD in simulation trajectories of the four bromodomains (ATAD2, BAZ2B, BRD2(1), CREBBP) are shown.


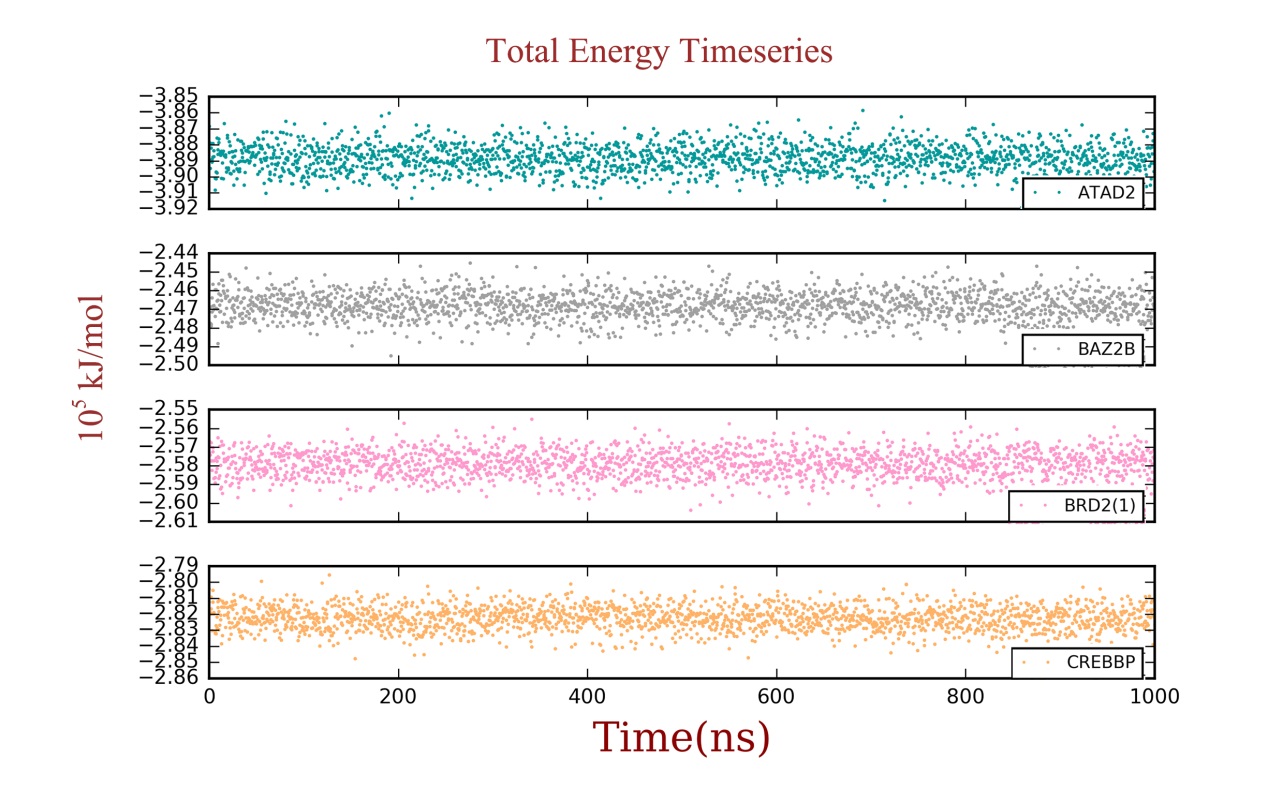


Figure D. The time series of the total energy in simulation trajectories of the four bromodomains (ATAD2, BAZ2B, BRD2(1), CREBBP) are shown.

**
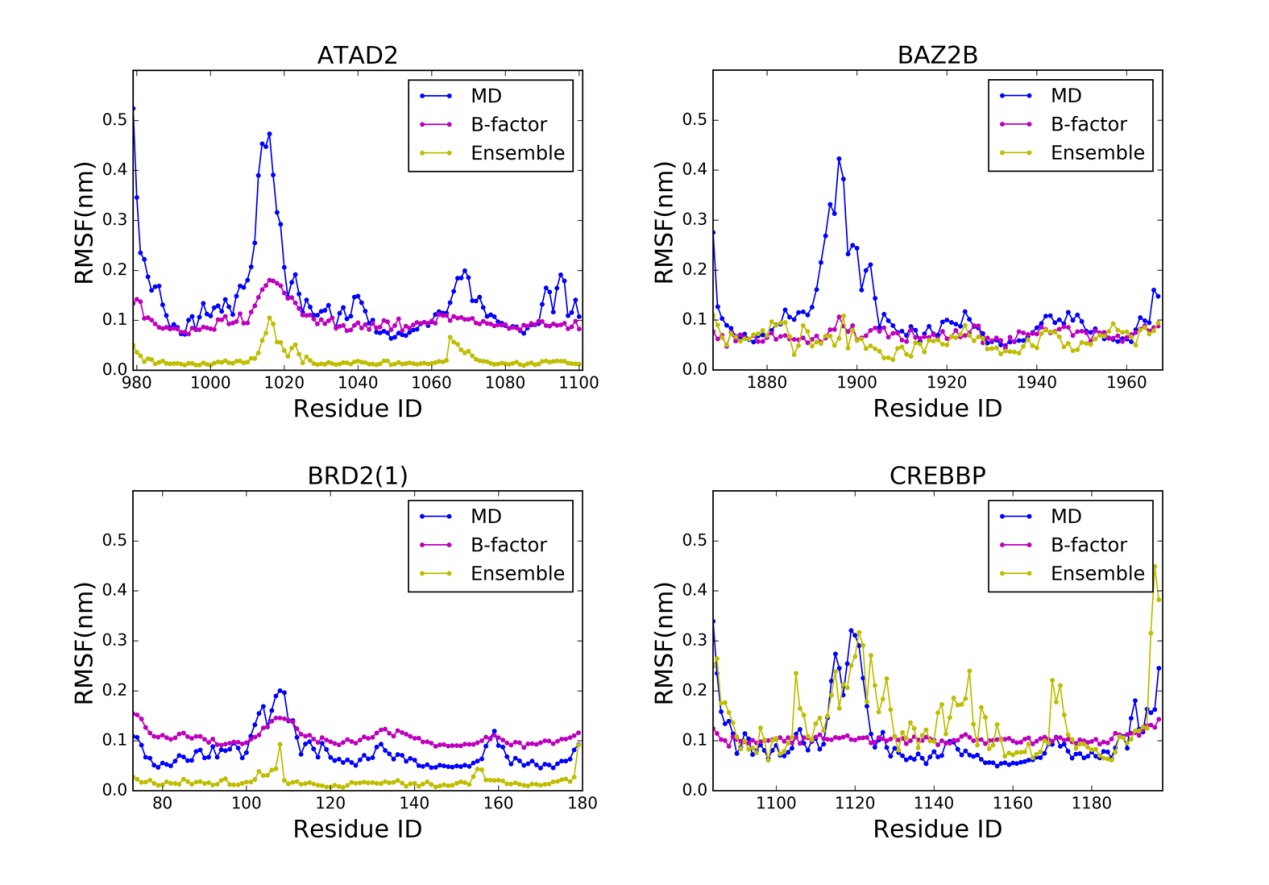
**

Figure E. The comparison of C-alpha RMSF calculated from 1000ns MD simulation, apo structure B-factor and RMSF of the crystal structure ensemble (chain A) of the four bromodomains (ATAD2, BAZ2B, BRD2(1), CREBBP).

| **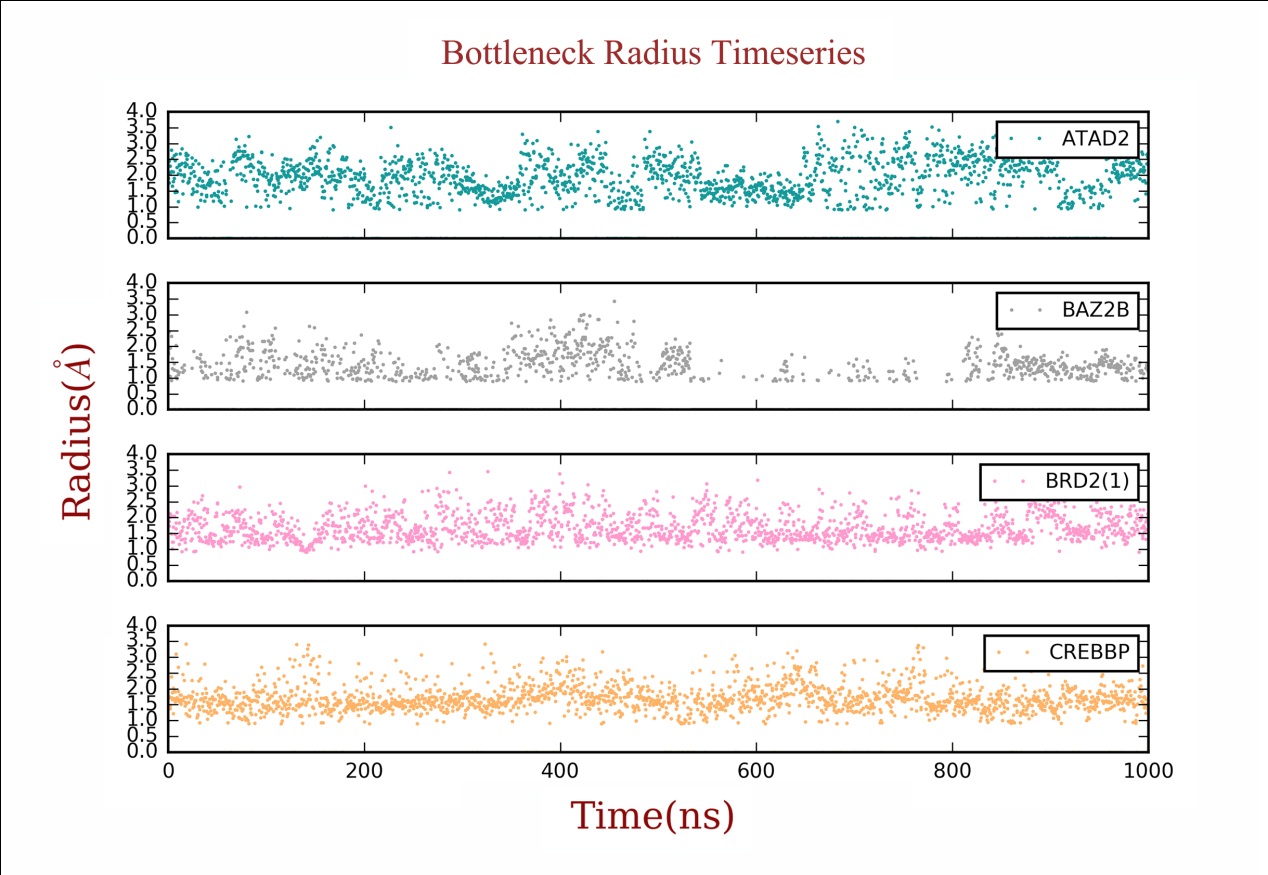** |
| --- |

Figure F. The time series of the bottleneck radius in simulation trajectories of the four bromodomains (ATAD2, BAZ2B, BRD2(1), CREBBP) are shown. When the bottleneck radius is less than 1 Å, the tunnel is not defined.


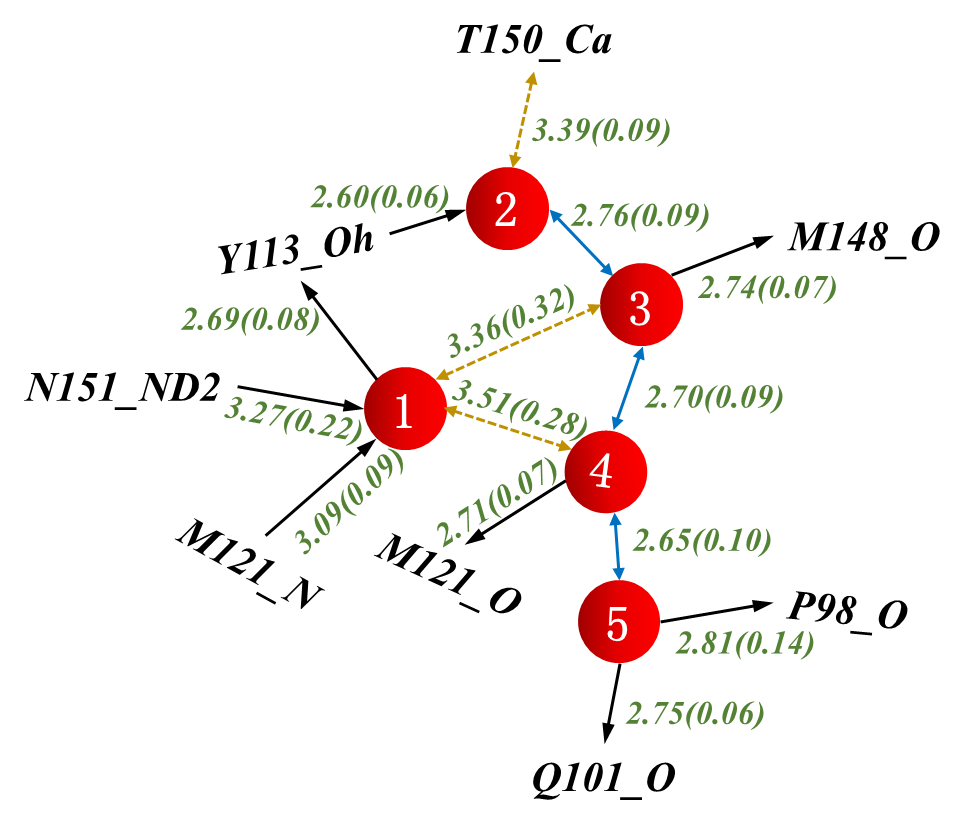


Figure G. The conserved water network at the bottom of bromodomain active site is shown. The five waters are labeled from 1 to 5 according to the H-bond chain and shown in red spheres, representing the water oxygen atom. The residue indexes come from BRD2(1) and the atom names can refer to Figure 2. The average and standard deviation within the bracelet are shown in green, unit: Å. The black arrows pointing from one atom to another atom show the donation of H-bond hydrogen. The blue arrows show the mutual H-bond donation or acceptation between the waters. The yellow arrows show possible interactions between atoms.


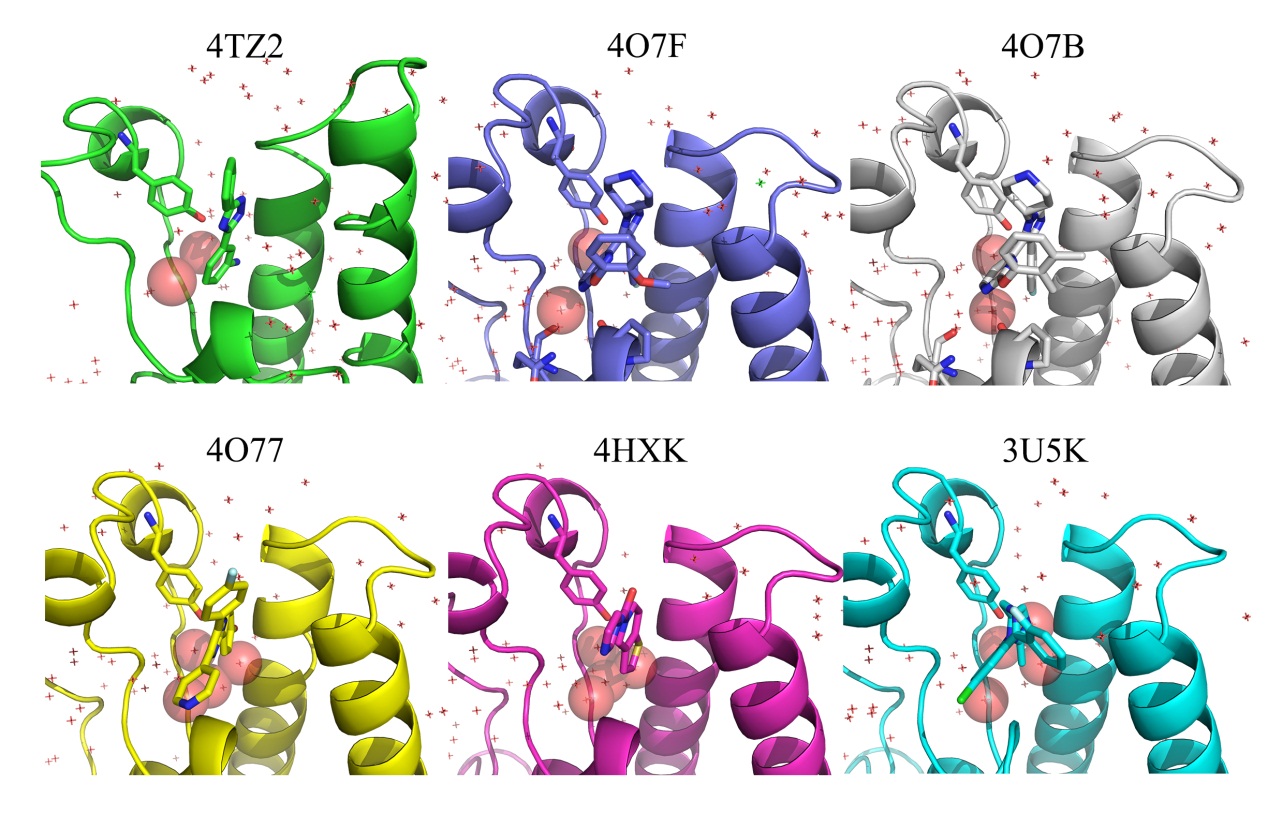


Figure H. The structures of bromodomains found to have replaced waters in the KAc pocket are shown. The proteins are shown in cartoon with the Tyr in PDY motif shown in sticks. The structural waters are shown in red spheres and the ligands are shown in sticks, other waters are shown in red dots.


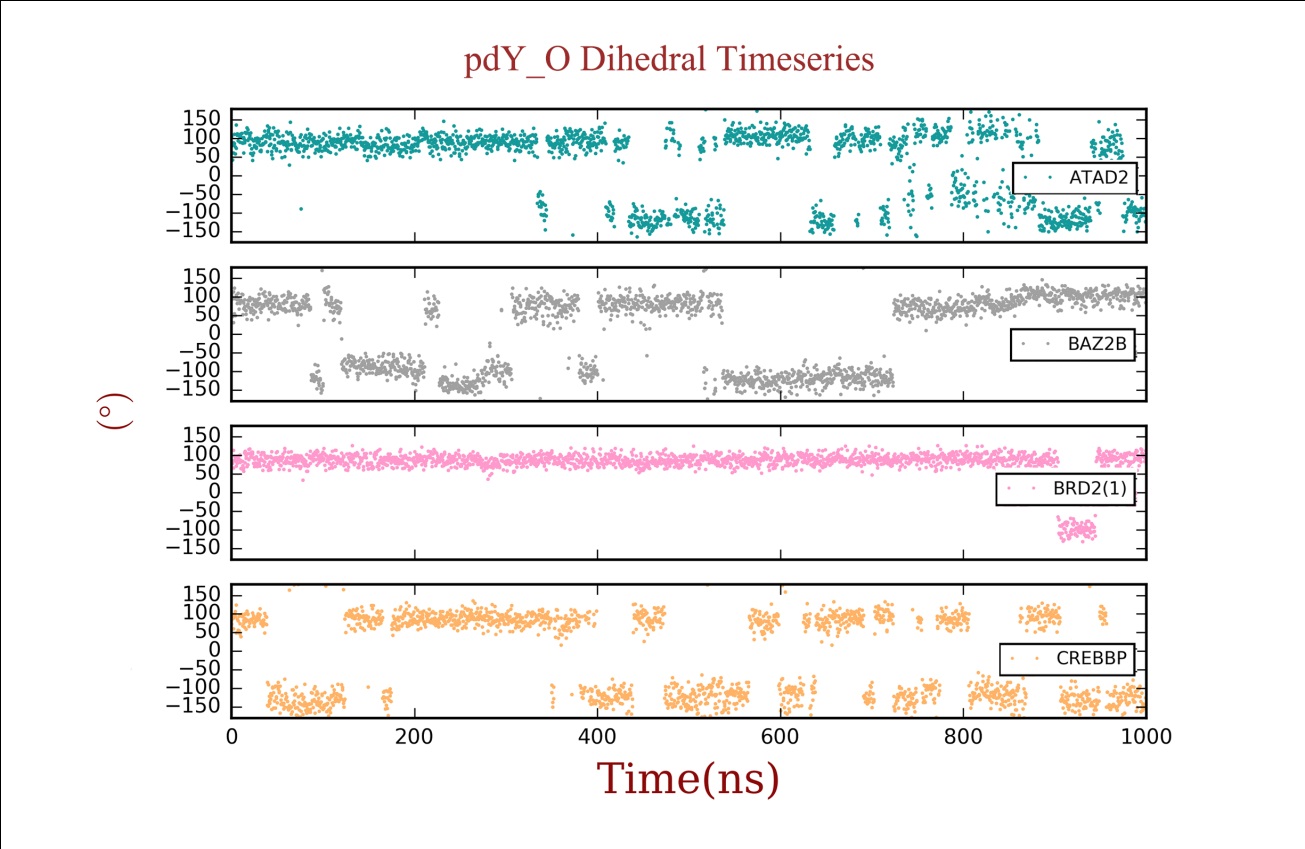


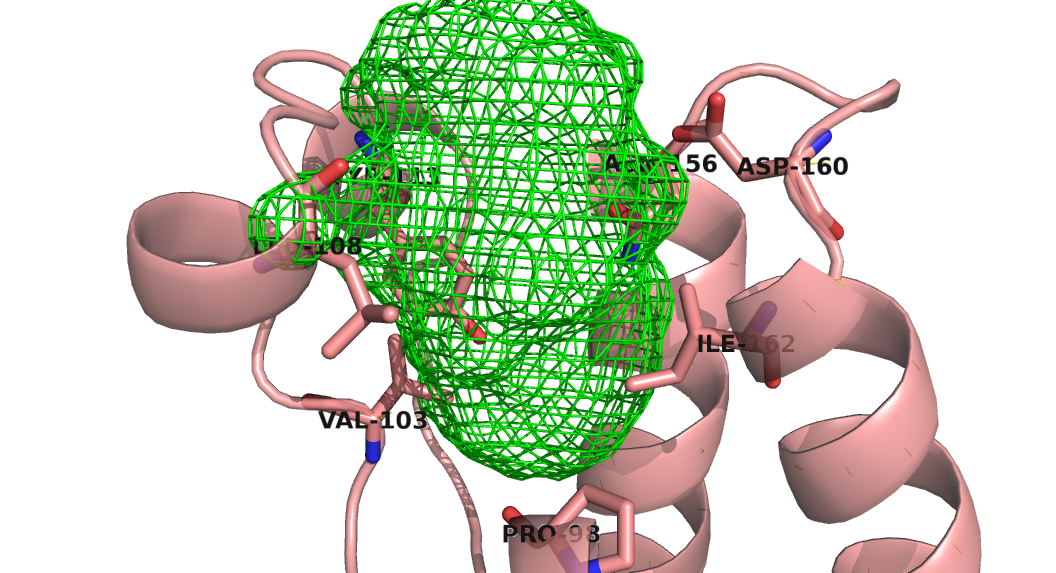
Figure I. The time series of the pdY_O dihedral angle in simulation trajectories of the four bromodomains (ATAD2, BAZ2B, BRD2(1), CREBBP) are shown. The dihedral angle hops between around 100º and -100º regions.

Figure J. The tunnel to BRD2(1) KAc binding pocket (PDB ID: 1X0J). The tunnel calculated by Caver 3.0 software is shown by green mesh. The tunnel bottleneck is formed by PVD, PDY motifs (V103 and Y113 in BRD2(1) ).
