# Supplementary material for: Protein dynamics and structural waters in bromodomains
Source: PLoS One. 2017 Oct 27;12(10):e0186570. doi: 10.1371/journal.pone.0186570 (PMC5659604; doi:10.1371/journal.pone.0186570)
Supplement: S2 File — A. Crystal and NMR structures used in MD simulation; B. Definition and residue numbers of structural waters; C. Observed cases of replaced (pink) and kept (blue) water molecules in crystal structures of the bromodomains; D. SPA results for water binding; E The percentage of the transitions of water 1–5; F. PDB codes of crystal structures used in the survey of water network. (DOCX) [file pone.0186570.s002.docx]

Supporting Information for:

Protein Dynamics and Structural Waters in Bromodomains

Xiaoxiao Zhang,^1^ Kai Chen,^1,2^ Yun-Dong Wu,^1,4*^ Olaf Wiest^1,3*^

1 Lab of Computational Chemistry and Drug Design, Laboratory of Chemical Genomics, Peking University Shenzhen Graduate School, Shenzhen 518055, China

2 Key Laboratory of Functional Molecular Engineering of Guangdong Province , School of Chemistry and Chemical Engineering, , South China University of Technology, Guangzhou 510640, China

3 Department of Chemistry and Biochemistry, University of Notre Dame, Notre Dame, Indiana 46556-5670, USA

4 College of Chemistry and Molecular Engineering, Peking University, Beijing 100871, China

owiest@nd.edu

Table A. Crystal and NMR structures used in MD simulation

Table B. Definition and residue numbers of structural waters

Table C. Observed cases of replaced (pink) and kept (blue) water molecules in

crystal structures of the bromodomains Table D. SPA results for water binding

Table E Percentage of the transitions of water 1-5

Table F. PDB codes of crystal structures used in the survey of water network

Table A. The crystal and NMR structures used in MD simulation are shown with corresponding PDB code, resolution and R-free parameters.

| Protein | PDB Code | Resolution | R Free |
| --- | --- | --- | --- |
| ATAD2 | 3DAI | 1.95 | 0.240 |
| BAZ2B | 3G0L | 2.03 | 0.211 |
| BRD2(1) | 1X0J | 1.80 | 0.213 |
| CREBBP | 3DWY | 1.98 | 0.219 |

These structures, as well as the structures listed in Table F, are accessible using the four-digit pdb code at <http://www.rcsb.org/pdb>.

Table B. The structural waters are defined based on distance matrix which is composed of the distances between each water oxygen coordinates to specific atoms of the protein. This table shows the conditions of defining a water molecule as one of the structural waters and the corresponding residue IDs in each bromodomain. The markers are defined by bromodomain sequence logo and atom names are based on Amber force field.

| Water | Marker | Atom | Distance(Å) | ATAD2 | BAZ2B | BRD2(1) | CREBBP |
| --- | --- | --- | --- | --- | --- | --- | --- |
| 1 | pMd | N | 2.5~4.0 | 1029 | 2105 | 121 | 1133 |
|  | N1 | ND2 | 2.5~4.0 | 1059 | 2135 | 151 | 1163 |
|  | pdY | OH | 2.5~4.5 | 1021 | 2097 | 113 | 1125 |
| 2 | pdY | OH | 2.5~3.5 | 1021 | 2097 | 113 | 1125 |
|  | N1p1 | CA | 2.5~5.0 | 1058 | 2134 | 150 | 1162 |
|  | V1 | O | 3.5~6.0 | 1056 | 2132 | 148 | 1160 |
| 3 | V1 | O | 2.5~3.5 | 1056 | 2132 | 148 | 1160 |
|  | N1 | CB | 2.5~5.0 | 1059 | 2135 | 151 | 1163 |
| 4 | pMd | O | 2.5~3.5 | 1029 | 2105 | 121 | 1133 |
|  | pmD | CA | 2.5~5.0 | 1030 | 2106 | 122 | 1134 |
|  | V1 | O | 3.5~6.0 | 1056 | 2132 | 148 | 1160 |
| 5 | wPf | O | 2.5~4.5 | 1008 | 2084 | 98 | 1110 |
|  | prep | O | 2.5~5.0 | 1011 | 2087 | 101 | 1113 |
|  | pMd | O | 2.5~6.0 | 1029 | 2105 | 121 | 1133 |

Table C. Observed cases of replaced (pink) and kept (blue) water molecules in crystal structures of the bromodomains.

| **Name** | **1** | **2** | **3** | **4** | **5** | **Observation** | **Affinity** |
| --- | --- | --- | --- | --- | --- | --- | --- |
| ATAD2_4TZ2 |  |  |  |  |  | Replaced by bulky rings | K_d_= 350 μM |
| BRD4(1)_3U5K |  |  |  |  |  | Water network distorted | μM |
| BRD4(1)_4HXK |  |  |  |  |  | Wat1 replaced by bulky ring | Not reported |
| BRD4(1)_4O77 |  |  |  |  |  | Wat1 replaced by –OH | IC_50_=2.5μM  IC_50_=20μM |
| BRD4(1)_4O7B |  |  |  |  |  | Wat1-3 replaced by bulky ring |  |
| BRD4(1)_4O7F |  |  |  |  |  | Wat1-3 replaced by bulky ring | IC_50_=13μM |
| PB1(5)_4Q0N  PB5_4q0n_A |  |  |  |  |  | Wat1-3 replaced by bulky ring | Not reported |
| PB1(5)_4Q0O |  |  |  |  |  | Wat1-3 replaced by bulky ring | Not reported |
| BRD4(1)_5I88 |  |  |  |  |  | Wat3 replaced by C=C | IC_50_=0.46μM |
| TAF1(2)_5I29 |  |  |  |  |  | Wat3 replaced by C=C | IC_50_=0.05μM |
| TAF1(2)_4YYN |  |  |  |  |  | Wat3 replaced by C=C | Kd=110μM |

Table D. The results calculated by SPA program using 5000 frames extracted from 1000ns MD simulation trajectories of the four bromodomains. WS stands for water sites 1-5 and yN bound water named as “N”. RANK stands for the ID of specific water site in 100 calculated clusters, the corresponding VDWsol, ELEsol stands for VDW and electrostatic interaction energies between the specific water and the solvent; VDWrec and ELErec stands for VDW and electrostatic interaction energies between the specific water and the receptor; T_TS and R_TS are calculated rotational and translational entropy change; and the TOTsys means the total interaction energy.

| WS | RANK | VDWsol | ELEsol | VDWrec | ELErec | T_TS | R_TS | TOTsys |
| --- | --- | --- | --- | --- | --- | --- | --- | --- |
| ATAD2 | | | | | | | | |
| 1 | 19 | 0.7 | -4.3 | 0.7 | -16.4 | 0.3 | 0.4 | -6.7 |
| 2 | 2 | 2.9 | -14.4 | -1.5 | -5.6 | 0.4 | 0.2 | -3.1 |
| 3 | 5 | 2.6 | -12.7 | -1.1 | -8.9 | 0.3 | 0.2 | -4.8 |
| 4 | 28 | 3.1 | -15.4 | -1.2 | -6.1 | 0.2 | 0.1 | -3.6 |
| 5 | 66 | 2.5 | -13.7 | -0.9 | -6.7 | 0.2 | 0.1 | -3.4 |
| N | 81 | 2.4 | -13.1 | -0.3 | -8.1 | 0.2 | 0.0 | -3.8 |
| BAZ2B | | | | | | | | |
| 1 | 13 | 1.0 | -5.4 | 0.4 | -16.5 | 0.3 | 0.3 | -7.3 |
| 2 | 83 | 2.8 | -12.9 | -1.6 | -7.8 | 0.2 | 0.3 | -4.4 |
| 3 | 16 | 2.7 | -13.5 | -1.4 | -6.1 | 0.3 | 0.2 | -3.1 |
| 4 | 43 | 2.7 | -13.7 | -1.3 | -7.2 | 0.2 | 0.2 | -4.0 |
| 5 | 60 | 2.0 | -9.8 | -0.5 | -11.8 | 0.2 | 0.1 | -5.6 |
| BRD2(1) | | | | | | | | |
| 1 | 4 | 0.6 | -6.6 | -0.2 | -13.6 | 0.5 | 0.5 | -6.5 |
| 2 | 2 | 2.5 | -13.5 | -0.6 | -9.8 | 0.6 | 0.6 | -5.6 |
| 3 | 1 | 3.2 | -13.9 | -1.2 | -9.4 | 0.6 | 0.6 | -5.3 |
| 4 | 3 | 3.3 | -14.6 | -0.9 | -7.1 | 0.5 | 0.7 | -3.6 |
| 5 | 8 | 2.4 | -10.9 | -0.9 | -8.9 | 0.4 | 0.4 | -3.8 |
| N | 29 | 2.6 | -15.7 | -1.2 | -3.9 | 0.3 | 0.2 | -2.4 |
| CREBBP | | | | | | | | |
| 1 | 2 | 0.8 | -5.1 | 0.5 | -15.7 | 0.5 | 0.4 | -6.6 |
| 2 | 5 | 2.3 | -12.8 | -1.0 | -8.5 | 0.5 | 0.2 | -4.8 |
| 3 | 89 | 2.2 | -10.1 | -1.3 | -9.5 | 0.3 | 0.5 | -4.5 |
| 4 | 27 | 3.2 | -14.3 | -1.3 | -6.4 | 0.3 | 0.2 | -3.3 |
| 5 | 18 | 1.3 | -6.2 | 0.4 | -15.0 | 0.4 | 0.4 | -6.3 |
| N | 20 | 2.5 | -15.6 | -0.6 | -4.3 | 0.4 | 0.1 | -2.3 |

Table E. The percentage of the transitions of water 1-5 to each other and the bulky waters are shown, the waters mainly flow into the bulky solvent.

|  | ATAD2 (%) | | | | | BAZ2B (%) | | | | | BRD2(1) (%) | | | | |
| --- | --- | --- | --- | --- | --- | --- | --- | --- | --- | --- | --- | --- | --- | --- | --- |
|  | **wat1** | **wat2** | **wat3** | **wat4** | **wat5** | **wat1** | **wat2** | **wat3** | **wat4** | **wat5** | **wat1** | **wat2** | **wat3** | **wat4** | **wat5** |
| **wat1** | **0.0** | **0.4** | **0.7** | **1.0** | **0.1** | **0.0** | **0.5** | **1.6** | **1.2** | **0.2** | **0.0** | **1.0** | **1.9** | **1.5** | **0.3** |
| **wat2** | **0.2** | **0.0** | **1.2** | **0.6** | **0.3** | **0.4** | **0.0** | **1.4** | **0.3** | **0.3** | **0.9** | **0.0** | **1.6** | **0.6** | **0.3** |
| **wat3** | **0.7** | **1.0** | **0.0** | **1.3** | **0.4** | **2.0** | **1.3** | **0.0** | **1.8** | **0.6** | **1.9** | **1.6** | **0.0** | **2.4** | **0.7** |
| **wat4** | **0.9** | **0.9** | **1.3** | **0.0** | **1.1** | **1.5** | **0.5** | **1.8** | **0.0** | **0.5** | **1.8** | **0.6** | **2.1** | **0.0** | **1.8** |
| **wat5** | **0.2** | **0.3** | **0.4** | **1.1** | **0.0** | **0.3** | **0.2** | **0.4** | **0.3** | **0.0** | **0.3** | **0.3** | **0.7** | **2.1** | **0.0** |
| **out** | **11.6** | **22.4** | **19.4** | **19.2** | **13.3** | **15.8** | **16.6** | **16.2** | **18.6** | **15.8** | **12.7** | **18.1** | **13.3** | **15.5** | **16.0** |
| CREBBP (%) | | | | |  |  |  |  |  |  |  |  |  |  |  |
| **wat1** | **wat2** | **wat3** | **wat4** | **wat5** |  |  |  |  |  |  |  |  |  |  |  |
| **0.0** | **0.6** | **1.1** | **0.6** | **0.2** |  |  |  |  |  |  |  |  |  |  |  |
| **0.7** | **0.0** | **0.6** | **0.2** | **0.0** |  |  |  |  |  |  |  |  |  |  |  |
| **1.2** | **0.6** | **0.0** | **0.4** | **0.2** |  |  |  |  |  |  |  |  |  |  |  |
| **0.9** | **0.2** | **0.4** | **0.0** | **0.1** |  |  |  |  |  |  |  |  |  |  |  |
| **0.2** | **0.1** | **0.1** | **0.1** | **0.0** |  |  |  |  |  |  |  |  |  |  |  |
| **25.5** | **21.6** | **19.4** | **13.9** | **10.9** |  |  |  |  |  |  |  |  |  |  |  |

Table F. PDB codes of the crystal structures used in the survey of water network and active site distance matrix is shown.

| Protein | Code | Protein | Code | Protein | Code | Protein | Code |
| --- | --- | --- | --- | --- | --- | --- | --- |
| ATAD2 | 4QSP | BAZ2B | 3Q2F | BAZ2B | 4QC3 | CREBBP | 3P1C |
| ATAD2 | 4QSQ | BAZ2B | 4CUP | BRD2(1) | 2YEK | CREBBP | 3P1D |
| ATAD2 | 4QSR | BAZ2B | 4CUR | BRD2(1) | 2YDW | CREBBP | 3P1E |
| ATAD2 | 4QSS | BAZ2B | 4CUS | BRD2(1) | 3AQA | CREBBP | 3P1F |
| ATAD2 | 4QST | BAZ2B | 4CUT | BRD2(1) | 4A9E | CREBBP | 3SVH |
| ATAD2 | 4QSU | BAZ2B | 4CUU | BRD2(1) | 4A9F | CREBBP | 4A9K |
| ATAD2 | 4QSV | BAZ2B | 4IR3 | BRD2(1) | 4A9H | CREBBP | 4NR4 |
| ATAD2 | 4QSW | BAZ2B | 4IR4 | BRD2(1) | 4A9J | CREBBP | 4NR5 |
| ATAD2 | 4QSX | BAZ2B | 4IR5 | BRD2(1) | 4A9M | CREBBP | 4NR6 |
| ATAD2 | 4QUT | BAZ2B | 4IR6 | BRD2(1) | 4A9N | CREBBP | 4NR7 |
| ATAD2 | 4QUU | BAZ2B | 4NR9 | BRD2(1) | 4A9O | CREBBP | 4NYR |
| ATAD2 | 4TTE | BAZ2B | 4NRA | BRD2(1) | 4AKN | CREBBP | 4NYW |
| ATAD2 | 4TU4 | BAZ2B | 4NRB | BRD2(1) | 4ALG | CREBBP | 4N3W |
| ATAD2 | 4TYL | BAZ2B | 4NRC | BRD2(1) | 4ALH | CREBBP | 4N4F |
| ATAD2 | 4TZ2 | BAZ2B | 4RVR | BRD2(1) | 4UYF | CREBBP | 4NYV |
| BAZ2B | 4QC1 | BAZ2B | 4QBM | BRD2(1) | 4UYH |  |  |
| BAZ2B | 4XUA | BAZ2B | 4XUB |  |  |  |  |
